# Supplementary material for: An annotated chromosome-scale reference genome for Eastern black-eared wheatear (Oenanthe melanoleuca)
Source: G3 (Bethesda). 2023 Apr 25;13(6):jkad088. doi: 10.1093/g3journal/jkad088 (PMC10234393; doi:10.1093/g3journal/jkad088)
Supplement: jkad088_Supplementary_Data [file jkad088_supplementary_data.zip › Figure-S1.pdf]

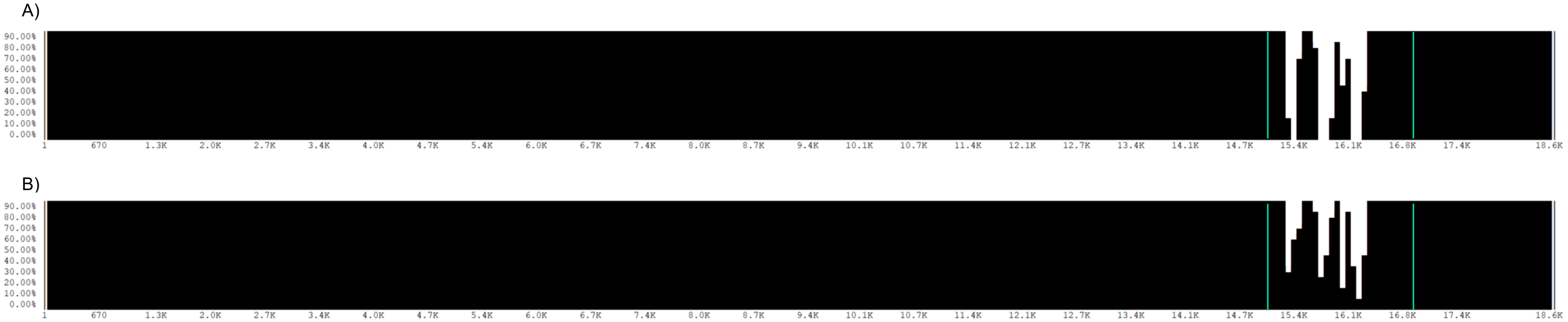

Figure S1. Illumina short read (A) and PacBio long-read (B) coverage along the MitoVGP assembly. Coverage is shown as percentage of highest read coverage in bins of 10%. The insertion in the MitoVGP assembly relative to the mitoFinder assembly and mitogenomes of Isabelline and Northern Wheatear is situated between the green demarkations.
