# Supplementary material for: An annotated chromosome-scale reference genome for Eastern black-eared wheatear (Oenanthe melanoleuca)
Source: G3 (Bethesda). 2023 Apr 25;13(6):jkad088. doi: 10.1093/g3journal/jkad088 (PMC10234393; doi:10.1093/g3journal/jkad088)
Supplement: jkad088_Supplementary_Data [file jkad088_supplementary_data.zip › List and captions of supplementary materials.pdf]

List and captions of supplementary materials

Figure S1. Illumina short read (A) and PacBio long-read (B) coverage along the MitoVGPassembly. Coverage is shown as percentage of highest read coverage in bins of 10%. The insertion in the MitoVGPassembly relative to the mitoFinderassembly and mitogenomes of Isabelline and Northern Wheatear is situated between the green demarkations.

Figure S2. Frequencies of GO terms in the *Oenanthe melanoleuca* reference genome annotation.

File S1. Library of consensus sequences of transposable elements and satellite DNA monomers for the species *Oenanthe melanoleuca* produced by RepeatModeler2 and RepeatExplorer2.

File S2. Repeat annotation of the *Oenanthe melanoleuca* genome assembly with divergence from consensus calculated with Kimura-2 parameter distance model and corrected for the presence of CpG sites.

Table S1. Summary table of the percentages of repeats annotated in the genome assembly of *Oenanthe melanoleuca*.
