# Supplementary figures and images for: An annotated chromosome-scale reference genome for Eastern black-eared wheatear (Oenanthe melanoleuca)
Source: G3 (Bethesda). 2023 Apr 25;13(6):jkad088. doi: 10.1093/g3journal/jkad088 (PMC10234393; doi:10.1093/g3journal/jkad088)

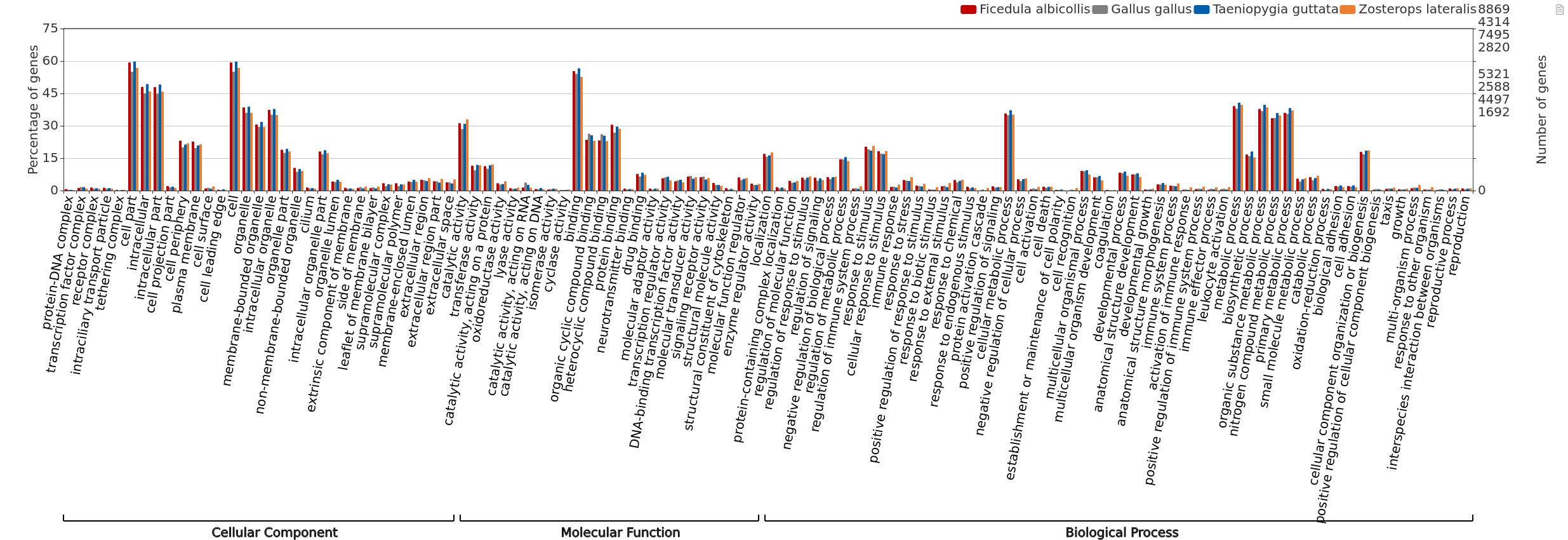

Supplement: jkad088_Supplementary_Data [file jkad088_supplementary_data.zip › Figure-S2_new.jpeg]
